# Supplementary material for: RNA virus attenuation by codon pair deoptimisation is an artefact of increases in CpG/UpA dinucleotide frequencies
Source: eLife. 2014 Dec 9;3:e04531. doi: 10.7554/eLife.04531 (PMC4383024; doi:10.7554/eLife.04531)
Supplement: Supplementary file 1. — Nucleotide sequences of mutants used in the study. Supplementary file 1.docx http://dx.doi.org/10.7488/ds/188. DOI: http://dx.doi.org/10.7554/eLife.04531.019 [file elife04531s001.doc]

Nucleotide sequences of mutants used in the study

*Region 1*

>R1_WT (W|W)

GTCGACTCCGTGGTGCCCGTCAACAATATCAAAGTCAACCTGCAAAGCATGGATGCGTATCATATTGAGGTCAATACCGGGAACCACCAGGGGGAAAAGA

TTTTTGCGTTCCAAATGCAGCCGGGGTTAGAGTCTGTTTTCAAGAGAACCCTTATGGGGGAGATTCTTAATTATTATGCACACTGGTCAGGGAGCATTAA

GCTGACATTCACATTTTGTGGATCGGCGATGGCAACTGGAAAACTCTTGTTAGCGTATTCACCACCAGGTGCTGATGTGCCCGCGACCAGGAAACAGGCG

ATGTTAGGCACACACATGATTTGGGATATCGGGCTTCAGTCGAGCTGTGTTTTGTGCATCCCATGGATAAGTCAGACACACTACCGGTTAGTGCAACAAG

ATGAATACACGAGTGCAGGCAATGTGACGTGTTGGTACCAAACAGGAATAGTGGTGCCCCCTGGCACTCCAAATAAGTGTGTAGTGCTTTGTTTTGCATC

AGCTTGTAATGATTTCTCAGTTCGAATGCTTAGGGACACCCCTTTCATCGGACAAACAGCACTGCTGCAAGGCGACACCGAAACGGCTATTGACAATGCA

ATCGCCAGGGTAGCAGATACGGTGGCGAGCGGTCCTAGTAATTCGACCAGTATCCCAGCACTCACAGCAGTTGAGACAGGTCACACGTCACAAGTCGAGC

CCAGCGATACAATGCAGACTAGACATGTCAAAAACTACCACTCGCGTTCTGAGTCAACCGTGGAAAACTTTCTAAGTCGCTCCGCTTGTGTGTACATCGA

AGAGTACTACACCAAGGACCAAGACAATGTTAATAGGTACATGTCGTGGACAATAAATGCCAGAAGAATGGTGCAATTGAGGAGAAAGTTTGAGCTGTTT

ACATACATGAGATTTGATATGGAAATCACGTTTGTAATCACAAGTAGACAACTACCTGGGACTAGCATAGCACAAGATATGCCGCCACTCACCCACCAGA

TCATGTACATACCACCAGGTGGCCCGGTACCAAACAGCGTAACAGATTTTGCGTGGCAGACATCAACAAACCCCAGCATTTTCTGGACAGAAGGAAACGC

GCCACCTCGCATGTCTATTCCATTCATCAGTATTGGCAATGCATATAGCAACTTCTATGACGGGTGGTCACACTTTTCCCAAAACGGTGTGTACGGATAC

AACGCCCTGAACAACATGGGCAAGCTGTACGCACGTCATGTTAAC

>R1_CDLR (P|P)

GTCGACTCCGTGGTCCCAGTAAACAATATAAAAGTCAACCTTCAAAGCATGGATGCATACCATATTGAAGTTAATACAGGCAATCACCAGGGTGAAAAAA

TCTTCGCGTTTCAAATGCAACCGGGACTGGAGTCAGTTTTCAAGAGAACGCTGATGGGGGAGATTCTCAACTATTATGCACACTGGTCAGGAAGCATCAA

GCTGACGTTCACATTCTGCGGGTCGGCCATGGCTACTGGCAAGCTGTTGTTAGCATATTCACCACCCGGTGCCGATGTGCCCGCAACAAGAAAACAGGCA

ATGTTGGGAACACACATGATTTGGGATATTGGTCTGCAGTCCAGCTGTGTTTTGTGTATCCCATGGATAAGTCAGACACACTACCGGTTAGTGCAACAAG

ATGAATATACCAGTGCCGGGAATGTGACGTGTTGGTACCAGACAGGAATAGTTGTGCCACCTGGCACCCCGAATAAGTGTGTCGTTCTTTGTTTTGCGTC

GGCATGTAATGATTTTTCAGTGCGCATGCTTAGGGACACCCCTTTCATCGGACAAACAGCACTGCTGCAAGGCGACACGGAAACAGCAATAGACAATGCT

ATCGCCAGGGTAGCAGATACAGTGGCGAGTGGTCCTAGTAATTCCACGAGCATCCCCGCTCTCACTGCAGTAGAGACTGGTCACACTTCGCAAGTTGAGC

CGAGTGATACGATGCAGACGAGACATGTTAAAAACTACCACTCTCGTTCGGAGTCAACTGTAGAAAACTTTCTCAGTCGATCCGCTTGTGTGTACATTGA

GGAGTACTACACGAAGGACCAAGACAATGTCAATAGGTACATGTCATGGACAATAAATGCCAGGAGGATGGTGCAATTGAGAAGAAAGTTTGAGCTGTTT

ACGTACATGAGATTTGACATGGAGATCACGTTCGTAATCACTAGTAGGCAATTGCCTGGCACTAGCATAGCACAAGATATGCCACCACTTACACACCAGA

TCATGTACATACCCCCAGGCGGCCCAGTACCAAACAGCGTCACAGATTTTGCGTGGCAAACATCAACAAACCCCAGCATCTTCTGGACAGAAGGAAACGC

ACCCCCACGGATGTCAATACCATTCATCAGCATTGGGAATGCGTATAGTAACTTTTATGACGGGTGGTCCCATTTTTCCCAAAACGGGGTTTACGGCTAC

AACGCGCTTAACAACATGGGCAAGCTGTACGCACGACATGTTAAC

>R1_CpG/UpA-Low (cu|cu))

GTCGACTCAGTGGTGCCAGTCAACAACATCAAAGTCAACCTGCAAAGCATGGATGCTTATCACATTGAGGTCAACACAGGGAACCACCAGGGGGAAAAGA

TTTTTGCTTTCCAAATGCAGCCTGGGTTGGAGTCTGTTTTCAAGAGAACCCTGATGGGGGAGATTCTGAATTATTATGCACACTGGTCAGGGAGCATCAA

GCTGACATTCACATTTTGTGGATCTGCCATGGCAACTGGAAAACTCTTGTTGGCTTATTCACCACCAGGTGCTGATGTGCCTGCAACCAGGAAACAGGCC

ATGTTGGGCACACACATGATTTGGGACATTGGGCTTCAGTCCAGCTGTGTTTTGTGCATCCCATGGATCAGTCAGACACACTACAGGTTGGTGCAACAAG

ATGAATACACAAGTGCAGGCAATGTGACATGTTGGTACCAAACAGGAATTGTGGTGCCCCCTGGCACTCCAAACAAGTGTGTTGTGCTTTGTTTTGCATC

AGCTTGCAATGATTTCTCAGTCAGGATGCTCAGGGACACCCCTTTCATTGGACAAACAGCACTGCTGCAAGGAGACACAGAAACAGCCATTGACAATGCA

ATTGCCAGGGTTGCAGACACTGTGGCAAGTGGTCCAAGCAATTCAACCAGCATCCCAGCACTCACAGCAGTTGAGACAGGTCACACCTCACAAGTGGAGC

CCAGTGACACAATGCAGACAAGACATGTCAAAAACTACCACTCCAGGTCTGAGTCAACTGTGGAAAACTTTCTCAGCAGGTCAGCTTGTGTGTACATTGA

AGAGTACTACACCAAGGACCAAGACAATGTCAACAGGTACATGTCCTGGACAATCAATGCCAGAAGAATGGTGCAATTGAGGAGAAAGTTTGAGCTGTTC

ACATACATGAGATTTGACATGGAAATCACCTTTGTGATCACAAGCAGACAACTCCCTGGGACAAGCATTGCACAAGACATGCCACCACTCACCCACCAGA

TCATGTACATTCCACCAGGTGGCCCAGTGCCAAACAGTGTCACAGATTTTGCCTGGCAGACATCAACAAACCCCAGCATTTTCTGGACAGAAGGAAATGC

CCCACCAAGGATGTCCATTCCATTCATCAGCATTGGCAATGCATACAGCAACTTCTATGATGGGTGGTCACACTTTTCCCAAAATGGTGTGTATGGATAC

AATGCCCTGAACAACATGGGCAAGCTGTATGCAAGACATGTTAAC

>R1-Max-U

GTCGACTCGGTGGTTCCTGTCAACAACATAAAAGTAAATTTACAAAGCATGGATGCCTACCACATAGAAGTCAACACTGGAAATCATCAAGGAGAAAAAA

TATTTGCCTTCCAGATGCAGCCGGGGTTAGAAAGTGTCTTCAAGCGCACGCTCATGGGAGAAATATTAAATTATTATGCACACTGGAGTGGCTCCATCAA

ACTCACCTTCACCTTCTGTGGGTCGGCCATGGCAACTGGAAAATTATTATTAGCCTACTCGCCGCCGGGTGCTGATGTTCCTGCCACCAGGAAACAAGCC

ATGCTTGGAACACACATGATCTGGGACATTGGTTTACAAAGCAGCTGTGTCCTCTGCATCCCCTGGATCTCCCAAACTCACTACCGCCTTGTTCAACAAG

ATGAATACACGTCGGCGGGCAATGTCACCTGCTGGTACCAAACTGGAATTGTTGTACCGCCGGGCACCCCCAACAAATGTGTTGTTCTCTGCTTTGCCTC

GGCCTGCAATGACTTCTCAGTTCGAATGCTTCGAGACACGCCCTTCATTGGACAAACAGCATTATTACAAGGAGACACGGAGACGGCGATTGATAATGCA

ATTGCTCGTGTGGCAGACACGGTGGCCTCGGGGCCCAGCAACAGCACCAGCATCCCGGCGCTGACAGCTGTAGAAACTGGACACACCAGCCAAGTAGAGC

CCAGCGACACCATGCAGACCAGGCATGTAAAAAACTACCACAGCCGCTCGGAAAGTACTGTAGAAAACTTCCTCAGCCGCTCGGCCTGTGTCTACATAGA

AGAATATTACACCAAGGACCAAGATAATGTCAACCGCTACATGTCCTGGACCATCAATGCAAGAAGAATGGTACAACTTCGAAGGAAATTTGAGCTCTTC

ACCTACATGAGATTTGACATGGAGATCACCTTTGTCATCACCAGCAGACAACTTCCTGGGACCAGCATTGCTCAAGACATGCCGCCGCTCACCCACCAGA

TCATGTACATCCCGCCGGGTGGTCCTGTTCCCAACTCGGTGACAGATTTTGCCTGGCAGACCAGCACCAACCCCAGCATCTTCTGGACAGAAGGAAATGC

GCCGCCGCGCATGTCCATCCCCTTCATCTCCATTGGAAATGCCTACAGCAACTTCTATGATGGCTGGAGCCACTTCTCCCAAAATGGTGTTTATGGCTAC

AACGCGCTGAACAACATGGGAAAACTCTACGCGCGCCATGTTAAC

>R1-Min-E

GTCGACTCCGTGGTGCCCGTCAACAATATCAAAGTCAATCTGCAAAGCATGGATGCGTATCATATTGAGGTCAATACCGGCAATCACCAGGGGGAAAAGA

TTTTTGCGTTTCAGATGCAACCGGGGTTAGAGTCTGTTTTCAAAAGAACCCTTATGGGGGAAATCCTTAATTACTATGCACATTGGTCAGGGAGCATTAA

GCTGACATTCACATTCTGTGGATCGGCGATGGCAACTGGCAAACTGCTGTTAGCGTATTCACCACCAGGTGCTGATGTGCCCGCGACAAGGAAACAGGCG

ATGTTAGGCACACACATGATTTGGGATATCGGACTGCAATCGAGCTGTGTGTTGTGCATCCCTTGGATAAGTCAGACACACTATCGGTTAGTGCAACAGG

ATGAGTACACGAGTGCAGGCAATGTGACGTGTTGGTATCAGACAGGCATAGTGGTGCCCCCTGGCACTCCAAATAAGTGTGTAGTGCTTTGTTTTGCATC

AGCTTGTAATGATTTCTCAGTTCGGATGCTTAGGGACACCCCTTTCATCGGTCAGACAGCACTGCTGCAAGGCGACACCGAAACGGCTATTGACAATGCA

ATCGCCAGGGTAGCAGATACGGTTGCGAGCGGTCCTAGTAATTCGACAAGTATCCCAGCACTCACAGCAGTTGAGACAGGTCACACGAGCCAAGTCGAGC

CCAGCGATACAATGCAGACTAGACATGTGAAAAACTATCATTCGCGGTCTGAGTCAACCGTTGAGAATTTCCTATCACGCTCCGCTTGTGTGTACATCGA

AGAGTATTACACCAAGGATCAGGACAATGTTAATAGGTACATGTCGTGGACCATAAATGCCAGAAGAATGGTGCAATTGAGGAGAAAGTTTGAACTGTTT

ACATACATGAGATTTGATATGGAAATCACGTTTGTAATCACAAGTAGACAACTACCTGGGACTAGCATAGCACAGGATATGCCGCCACTCACCCACCAAA

TCATGTACATACCACCAGGGGGACCGGTACCAAACAGCGTAACAGATTTTGCGTGGCAGACATCAACAAACCCCAGCATTTTTTGGACAGAGGGAAACGC

GCCACCTCGCATGTCTATTCCATTCATCAGTATTGGCAATGCATATAGCAATTTCTATGACGGGTGGTCACACTTTTCCCAAAACGGTGTGTACGGATAC

AACGCCCTGAACAACATGGGCAAACTGTACGCACGGCATGTTAAC

>R1-Min-H

GTCGACTCCGTCGTTCCTGTGAACAATATTAAGGTTAATTTACAATCCATGGATGCCTACCATATCGAAGTAAACACTGGAAATCATCAAGGCGAAAAGA

TTTTCGCCTTCCAGATGCAGCCCGGTTTAGAAAGTGTCTTCAAGAGAACGCTGATGGGAGAAATTTTAAATTATTATGCACATTGGAGTGGTTCGATTAA

ATTAACGTTTACCTTCTGCGGTAGCGCTATGGCCACGGGTAAGTTATTATTAGCGTATAGTCCGCCGGGCGCAGATGTTCCCGCTACCCGTAAACAAGCG

ATGCTCGGTACGCATATGATTTGGGACATCGGACTGCAAAGTAGTTGCGTTTTATGCATACCGTGGATTTCCCAGACGCATTATCGCCTCGTACAACAGG

ATGAATATACGAGCGCCGGTAACGTCACCTGCTGGTACCAGACCGGTATCGTCGTTCCCCCTGGGACCCCCAACAAGTGCGTCGTACTGTGTTTTGCATC

GGCCTGCAACGACTTCAGCGTTCGAATGTTACGCGACACGCCGTTCATCGGACAGACGGCGTTACTGCAAGGGGACACAGAAACCGCTATTGATAACGCC

ATCGCGCGTGTGGCCGATACTGTAGCCTCGGGACCTAGCAATTCGACCAGCATCCCGGCGCTTACCGCTGTCGAAACTGGCCACACGTCGCAAGTCGAAC

CCTCAGATACCATGCAGACTCGCCATGTTAAAAATTACCACTCGAGGTCCGAATCCACGGTAGAAAACTTCCTCAGCCGCTCGGCGTGCGTCTACATCGA

AGAATATTACACGAAGGATCAAGATAACGTCAACCGCTACATGTCCTGGACAATAAATGCACGCCGCATGGTGCAATTGCGCAGGAAGTTCGAACTGTTT

ACCTACATGAGGTTCGACATGGAAATTACGTTCGTAATCACCAGCAGACAATTGCCCGGCACCTCCATTGCTCAAGACATGCCGCCGCTCACCCATCAAA

TTATGTACATCCCTCCCGGCGGACCCGTACCCAACAGCGTAACCGACTTCGCATGGCAAACGTCCACGAATCCCAGCATATTTTGGACCGAAGGTAATGC

GCCGCCGCGCATGTCCATTCCTTTTATCTCGATCGGAAATGCTTATAGCAACTTCTACGACGGATGGTCGCATTTTTCGCAAAATGGTGTTTACGGGTATAACGCTCTTAACAATATGGGAAAACTCTACGCTAGGCATGTTAAC

>R1-Min-U

GTCGACTCCGTGGTGCCCGTCAACAATATCAAAGTCAACCTGCAAAGCATGGATGCGTATCATATTGAGGTCAATACCGGGAACCACCAGGGCGAAAAGA

TTTTTGCGTTCCAAATGCAGCCGGGGCTCGAATCTGTTTTCAAACGCACCCTTATGGGCGAAATTCTTAACTATTATGCACACTGGTCAGGGAGCATTAA

GCTGACATTCACATTTTGTGGATCGGCGATGGCAACTGGAAAACTGTTATTAGCGTATTCACCACCAGGTGCTGATGTGCCCGCGACCAGGAAACAGGCG

ATGTTAGGCACACACATGATTTGGGATATCGGGCTTCAGTCTAGCTGTGTTTTGTGCATACCGTGGATAAGTCAGACGCATTACCGGTTAGTGCAACAGG

ATGAATACACTAGCGCAGGCAACGTAACGTGTTGGTACCAAACAGGAATAGTGGTACCGCCCGGTACTCCAAATAAGTGTGTAGTGCTTTGTTTTGCATC

AGCTTGTAATGATTTCTCAGTTCGAATGCTTAGGGACACCCCTTTCATCGGACAAACAGCACTGTTACAAGGCGACACCGAAACGGCTATCGATAATGCA

ATCGCCAGGGTAGCAGATACGGTGGCGAGCGGTCCTAGTAATTCGACCAGTATCCCAGCACTCACCGCCGTAGAGACAGGTCACACGTCACAAGTCGAGC

CCAGCGATACAATGCAGACTAGGCATGTTAAGAATTACCACTCGCGTTCTGAGTCAACCGTGGAAAACTTTCTAAGTAGGTCCGCTTGTGTGTACATCGA

AGAGTATTACACCAAGGACCAAGACAATGTTAATAGGTACATGTCGTGGACAATAAATGCCAGAAGAATGGTGCAATTGAGGAGAAAGTTTGAGCTGTTT

ACGTACATGAGATTTGATATGGAAATCACGTTTGTAATTACGAGTAGACAACTACCTGGGACTAGCATAGCACAAGATATGCCGCCACTTACGCACCAGA

TCATGTACATACCGCCAGGTGGCCCGGTACCAAACAGCGTTACGGATTTTGCGTGGCAGACAAGTACGAACCCCAGCATTTTCTGGACAGAAGGAAACGC

GCCACCTCGTATGTCTATTCCATTCATCAGTATCGGAAATGCGTATAGCAACTTCTACGATGGGTGGTCACACTTTTCCCAAAACGGTGTGTACGGGTAT

AACGCTCTGAACAACATGGGCAAGCTATACGCACGTCATGTTAAC

*Region 2*

>R2_WT (W|W)

GAATTCGCCGTTGCTATGATGAAGAGAAACTCAAGTACAGTGAAGACTGAGTATGGTGAGTTTACTATGCTGGGCATCTATGACAAGTGGGCCGTTTTGC

CACGCCATGCTAAACCTGGACCAACCATCCTGATGAATGACCAAGAGGTCGGCGTGTTAGACGCCAAGGAACTAGTGGACAAGGATGGCACTAACCTGGA

GCTGACACTACTCAAGTTAAACCGGAATGAGAAGTTCAGAGACATCAGAGGCTTCTTGGCTAAGGAGGAAGTGGAAGTCAACGAGGCTGTGCTGGCAATA

AACACTAGCAAGTTTCCTAACATGTACATTCCAGTAGGGCAGGTTACAGATTACGGCTTCCTAAACCTGGGTGGTACACCCACCAAAAGAATGCTTATGT

ATAACTTCCCCACAAGAGCAGGCCAGTGTGGCGGGGTACTCATGTCCACTGGCAAAGTTTTGGGAATCCATGTTGGTGGAAATGGCCATCAAGGCTTCTC

AGCAGCACTTCTCAAACACTACTTTAATGATGAACAAGGAGAGATTGAGTTCATTGAGAGTTCAAAGGAAGCAGGGTTCCCAATCATTAACGCACCCAGT

AAAACCAAGCTGGAGCCAAGTGTCTTCCACCAAGTATTTGAAGGCAACAAAGAGCCAGCAGTCCTCAGGAACAGTGACCCACGTCTCAAAGCTAATTTCG

AGGAGGCCATCTTTTCCAAATACATTGGGAATGTCAACACACACATAGATGAATACATGTTGGAGGCTGTTGACCATTATGCCGGACAATTGGCCACCCT

AGATATCAGCACTGAACCAATGAAGTTGGAGGATGCTGTGTACGGTACTGAAGGCCTTGAAGCTCTTGACTTAACAACAAGTGCAGGCTACCCCTATGTC

GCACTGGGTATCAAGAAGAGAGACATCCTCTCGAAGAAGACCAAGGACCTGACCAAGCTGAAAGAGTGCATGGATAAGTATGGCCTGAATCTACCAATGG

TGACATACGTGAAAGATGAACTCAGATCT

>R2_CDLR (P|P)

GAATTCGCAGTGGCTATGATGAAGAGAAACTCAAGCACTGTGAAGACTGAGTATGGTGAGTTCACAATGCTTGGCATCTATGACAAGTGGGCAGTCTTGC

CACGCCATGCCAAACCTGGACCAACCATTCTGATGAATGACCAAGAGGTTGGCGTTCTAGACGCTAAGGAACTAGTAGACAAGGATGGTACAAACCTGGA

ACTCACACTCCTCAAGTTAAACCGCAATGAGAAGTTCAGAGACATCAGAGGCTTCTTGGCAAAGGAAGAAGTGGAGGTCAACGAGGCCGTCCTCGCCATA

AACACTAGTAAATTTCCCAACATGTACATACCTGTAGGCCAGGTTACAGATTACGGCTTCCTGAACCTGGGTGGCACACCCACCAAAAGAATGCTTATGT

ATAACTTCCCCACCAGAGCCGGGCAGTGTGGTGGGGTGCTGATGTCTACAGGGAAAGTTTTGGGCATCCATGTGGGAGGAAATGGCCATCAAGGTTTCTC

AGCAGCACTCCTGAAACACTACTTTAATGATGAGCAAGGCGAGATTGAGTTCATTGAGAGCTCAAAGGAAGCAGGGTTCCCTATCATTAACGCCCCTAGT

AAGACCAAGCTTGAGCCAAGTGTCTTCCACCAAGTTTTTGAAGGTAACAAAGAACCTGCAGTACTTAGAAATAGTGACCCACGCCTGAAAGCTAACTTTG

AGGAGGCCATATTCTCCAAGTACATCGGGAATGTCAACACTCACATTGATGAATACATGTTGGAGGCTGTTGACCATTATGCAGGTCAGTTGGCTACACT

GGACATCAGCACTGAGCCAATGAAGTTGGAGGATGCTGTGTACGGCACAGAAGGACTGGAAGCACTCGATTTAACAACGAGTGCCGGCTACCCCTATGTA

GCACTGGGGATCAAGAAGAGAGATATTTTGTCAAAAAAGACCAAGGACCTCACCAAGCTAAAAGAGTGTATGGACAAATATGGCCTCAATCTACCAATGG

TGACATACGTGAAAGATGAACTCAGATCT

>R2_CpG/UpA-Low (cu|cu)

GAATTCGCTGTTGCCATGATGAAGAGAAACTCAAGCACAGTGAAGACTGAGTATGGTGAGTTCACCATGCTGGGCATCTATGACAAGTGGGCAGTTTTGC

CAAGGCATGCCAAACCTGGACCAACCATCCTGATGAATGACCAAGAGGTTGGGGTGTTGGATGCCAAGGAACTGGTGGACAAGGATGGCACCAACCTGGA

GCTGACACTTCTCAAGTTGAACAGAAATGAGAAGTTCAGAGACATCAGAGGCTTCTTGGCCAAGGAGGAAGTGGAAGTCAATGAGGCTGTGCTGGCAATC

AACACCAGCAAGTTTCCCAACATGTACATTCCAGTGGGGCAGGTGACAGATTATGGCTTCCTGAACCTGGGTGGAACACCCACCAAAAGAATGCTCATGT

ACAACTTCCCCACAAGAGCAGGCCAGTGTGGAGGGGTTCTCATGTCCACTGGCAAAGTTTTGGGAATCCATGTTGGTGGAAATGGCCATCAAGGCTTCTC

AGCAGCACTTCTCAAACACTACTTCAATGATGAACAAGGAGAGATTGAGTTCATTGAGAGTTCAAAGGAAGCAGGGTTCCCAATCATCAATGCACCCAGC

AAAACCAAGCTGGAGCCAAGTGTCTTCCACCAAGTGTTTGAAGGCAACAAAGAGCCAGCAGTCCTCAGGAACAGTGACCCAAGGCTCAAAGCCAATTTTG

AGGAGGCCATCTTTTCCAAATACATTGGGAATGTCAACACACACATTGATGAATACATGTTGGAGGCTGTTGACCATTATGCAGGACAATTGGCCACCCT

GGACATCAGCACTGAACCAATGAAGTTGGAGGATGCTGTGTATGGCACTGAAGGCCTTGAAGCTCTTGACTTGACAACAAGTGCAGGCTACCCCTATGTG

GCACTGGGGATCAAGAAGAGAGACATCCTCTCAAAGAAGACCAAGGACCTGACCAAGCTGAAAGAGTGCATGGACAAGTATGGCCTGAATCTCCCAATGG

TGACATATGTGAAAGATGAACTCAGATCT

>R2-Max-U

GAATTCGCTGTGGCCATGATGAAAAGAAACAGCAGCACTGTAAAAACAGAATATGGAGAGTTCACCATGCTGGGCATCTATGACAAGTGGGCTGTTCTTC

CGCGCCATGCCAAGCCTGGGCCCACCATTTTAATGAATGATCAAGAAGTTGGTGTTTTAGATGCCAAGGAGCTGGTGGACAAAGATGGCACCAACCTGGA

GCTCACCTTATTAAAATTAAATAGAAATGAAAAGTTCAGAGACATCCGCGGCTTCTTGGCCAAGGAAGAAGTAGAAGTAAATGAAGCTGTTTTAGCCATC

AACACCAGCAAATTTCCCAACATGTACATCCCTGTTGGACAAGTAACAGACTACGGCTTCTTAAATTTAGGTGGCACCCCCACCAAGCGCATGCTCATGT

ACAACTTCCCCACGCGGGCGGGCCAGTGTGGTGGTGTTTTAATGTCCACTGGAAAAGTTCTTGGAATCCATGTTGGTGGAAATGGACATCAAGGCTTCTC

GGCGGCGCTGTTAAAACACTACTTCAATGATGAACAAGGAGAAATAGAATTTATAGAAAGCAGCAAGGAGGCGGGCTTCCCCATCATCAATGCACCCAGC

AAGACCAAGCTGGAGCCGTCGGTCTTCCATCAAGTATTTGAAGGAAATAAAGAACCAGCTGTTCTTCGAAACAGTGACCCGCGCCTCAAAGCCAACTTTG

AAGAAGCCATCTTCTCCAAATATATTGGAAATGTCAACACTCATATTGATGAATACATGTTAGAAGCTGTTGACCACTATGCAGGACAGCTGGCCACGCT

GGACATCTCCACAGAACCCATGAAATTAGAAGATGCTGTTTATGGAACAGAAGGATTAGAGGCGCTGGACCTCACCACGTCGGCGGGCTACCCCTATGTG

GCGCTGGGCATCAAGAAAAGAGACATCCTCAGCAAGAAGACCAAGGACCTCACCAAATTAAAAGAATGTATGGACAAATATGGCCTCAATTTACCCATGG

TCACCTATGTAAAAGATGAGCTGAGATCT

>R2-Min-E

GAATTCGCCGTTGCTATGATGAAGAGAAACTCAAGTACAGTGAAGACTGAGTATGGTGAGTTTACTATGCTGGGCATCTATGACAAATGGGCCGTTTTGC

CACGCCATGCTAAACCTGGACCAACAATCCTGATGAATGATCAGGAAGTCGGCGTGTTAGACGCCAAAGAGCTAGTTGACAAGGATGGCACTAATCTGGA

ACTGACACTACTGAAATTAAATCGCAATGAGAAATTCAGAGACATCAGGGGGTTTCTGGCTAAGGAGGAAGTGGAAGTCAACGAGGCTGTGCTGGCAATA

AACACTAGCAAATTCCCTAACATGTACATTCCAGTAGGCCAGGTTACAGATTACGGGTTCCTAAATCTGGGTGGTACACCCACCAAAAGAATGCTTATGT

ATAATTTCCCCACAAGAGCAGGTCAGTGTGGCGGGGTACTGATGAGCACTGGCAAAGTTTTGGGAATCCATGTGGGGGGGAATGGGCATCAGGGGTTCTC

AGCAGCACTTCTGAAACATTACTTTAATGATGAACAGGGGGAAATTGAGTTCATTGAGAGTTCAAAAGAGGCAGGGTTCCCAATCATTAACGCACCCAGT

AAAACCAAGCTTGAGCCAAGTGTCTTTCACCAGGTATTTGAGGGAAACAAAGAGCCAGCAGTCCTCAGGAACTCTGACCCACGTCTGAAAGCTAATTTCG

AGGAAGCCATTTTCTCCAAATACATTGGCAATGTGAACACTCACATAGATGAGTACATGCTTGAGGCTGTTGATCACTATGCCGGACAATTGGCCACCCT

AGATATCAGCACTGAACCAATGAAGCTTGAGGATGCTGTGTACGGTACTGAGGGACTTGAGGCTCTTGACTTAACAACAAGTGCAGGGTATCCCTATGTC

GCACTGGGTATCAAAAAGAGAGACATCCTCTCGAAAAAAACCAAGGATCTGACCAAACTGAAAGAGTGCATGGATAAGTATGGACTGAATCTACCAATGG

TGACATACGTGAAAGATGAACTGAGATCT

>R2-Min-U

GAATTCGCCGTAGCTATGATGAAGAGAAATTCGAGTACAGTGAAGACTGAATACGGTGAGTTTACTATGCTGGGCATCTATGACAAATGGGCCGTTTTGC

CACGCCATGCGAAACCTGGACCAACCATCCTGATGAATGACCAAGAGGTCGGCGTGTTAGACGCCAAGGAACTAGTGGACAAGGATGGCACTAATCTGGA

ACTGACACTACTCAAGTTAAACCGGAATGAGAAGTTCAGAGACATCCGCGGATTCCTCGCAAAGGAGGAAGTGGAAGTCAACGAGGCTGTGCTCGCAATA

AACACTAGCAAGTTTCCTAACATGTACATTCCAGTAGGGCAGGTTACCGATTACGGCTTCCTAAACCTGGGTGGTACACCGACCAAACGTATGCTTATGT

ATAATTTCCCTACGAGAGCAGGCCAGTGTGGCGGGGTACTCATGAGCACTGGCAAAGTTTTGGGAATCCACGTAGGTGGAAATGGCCATCAAGGCTTCTC

AGCAGCACTTCTCAAACACTACTTTAATGATGAACAAGGAGAGATCGAGTTCATCGAAAGTTCAAAGGAAGCAGGGTTCCCAATCATTAACGCACCCAGT

AAAACCAAGCTGGAGCCAAGTGTCTTCCACCAGGTATTTGAAGGCAACAAAGAGCCAGCAGTCCTCAGGAACAGTGATCCGCGTCTCAAAGCTAATTTCG

AGGAGGCCATCTTTTCCAAATACATTGGGAATGTCAACACACACATAGATGAATACATGTTGGAGGCTGTTGACCATTACGCCGGACAATTGGCCACCCT

AGATATCAGCACTGAACCAATGAAGTTGGAGGATGCTGTGTACGGTACTGAAGGCCTCGAAGCTCTCGATCTGACAACAAGTGCAGGCTACCCCTATGTC

GCACTGGGTATCAAAAAAAGAGACATCCTCTCGAAAAAAACTAAGGACCTGACCAAGCTGAAAGAGTGCATGGATAAGTACGGACTGAATCTACCAATGG

TGACATACGTGAAAGATGAACTCAGATCT

>R2-Min-H

GAATTCGCCGTTGCCATGATGAAGCGCAATTCGTCCACTGTAAAGACCGAATACGGCGAATTTACGATGCTGGGCATCTACGACAAGTGGGCCGTACTGC

CTCGCCACGCTAAACCTGGGCCCACGATTTTAATGAATGATCAAGAAGTAGGTGTTCTCGACGCTAAAGAGCTAGTAGATAAGGACGGTACCAACCTCGA

ACTCACCTTATTAAAATTAAACAGAAATGAAAAATTTAGAGATATCCGCGGATTCTTGGCCAAGGAAGAAGTAGAAGTCAACGAGGCTGTTCTCGCAATT

AACACCAGCAAATTCCCGAATATGTACATCCCGGTCGGACAGGTCACAGATTATGGATTTCTGAATCTGGGCGGCACCCCCACGAAGCGTATGCTCATGT

ACAACTTCCCGACTAGGGCGGGCCAATGTGGGGGCGTACTCATGAGTACCGGTAAAGTACTCGGTATACATGTTGGTGGTAACGGCCACCAGGGGTTCTC

GGCGGCGTTACTCAAACATTATTTTAATGATGAACAAGGCGAAATAGAATTTATAGAAAGCAGCAAAGAGGCGGGCTTTCCCATCATAAATGCTCCTTCG

AAAACCAAGCTCGAGCCCAGCGTATTCCACCAGGTATTTGAAGGAAATAAAGAACCGGCTGTTCTTCGAAATTCAGACCCCCGCCTCAAAGCCAACTTCG

AAGAGGCGATCTTTTCCAAGTACATCGGTAACGTCAACACGCATATTGATGAATACATGTTAGAAGCCGTAGACCACTACGCCGGACAACTGGCGACACT

CGACATCAGTACCGAACCGATGAAATTAGAAGACGCCGTCTACGGTACAGAAGGCCTCGAAGCGCTCGATCTCACCACTAGCGCCGGCTACCCCTACGTG

GCCCTCGGCATCAAGAAGAGAGACATCCTCAGCAAAAAAACGAAAGACCTCACCAAGTTAAAAGAATGTATGGATAAATATGGCCTCAATCTTCCTATGG

TTACGTACGTTAAAGATGAACTGAGATCT
